# Improving Referral and Continuity of Care Through Structured Outpatient Disposition Planning Enabled by Electronic Referrals: A Quality Improvement Study

**DOI:** 10.7759/cureus.100727

**Published:** 2026-01-04

**Authors:** Rabih Abou Leila

**Affiliations:** 1 Family Medicine, International Medical Centre, Jeddah, SAU

**Keywords:** ambulatory care, care coordination, closed-loop communication, continuity of care, electronic medical record (emr), electronic referral system, outpatient clinics, patient safety, quality improvement, referral management

## Abstract

Background

Outpatient visit closure in our institution lacked a standardized disposition process, resulting in referral and follow-up decisions that were frequently communicated verbally and not reliably documented or operationalized in the electronic medical record (EMR). This led to poor visibility of inter-clinic outpatient referrals, fragmented continuity of care, and limited operational oversight. This lack of structured disposition planning introduced risks of delayed diagnoses, duplicated care, and unplanned external referral.

Methods

A quality improvement intervention was implemented in a tertiary hospital to redesign outpatient disposition planning by embedding a standardized inter-clinic outpatient referral order within the EMR. Referral entry at visit closure triggered real-time operational follow-up by a centralized referral coordination team. The primary outcome was the number of documented inter-clinic outpatient referrals per week. Secondary outcomes included referral outcomes and time from referral entry to first patient contact. Data were analyzed over a 35-week post-implementation period.

Results

Baseline referral documentation averaged fewer than two referrals per week. Following implementation, the number of weekly documented referrals increased rapidly and was sustained at a mean of over 800 referrals per week. A total of 15,891 inter-clinic outpatient referrals were recorded during the study period. Of these, 8,423 referrals (53.0%) were new consultations and 7,093 referrals (47.0%) were follow-up referrals. Referral outcomes showed that 3,726 referrals (23.4%) resulted in scheduled outpatient appointments and 7,948 referrals (50.0%) resulted in same-day access or direct admission, yielding 11,674 referrals (73.5%) with a completed next step. Referrals that did not progress included 3,006 patient cancellations (18.9%), 581 referrals with unsuccessful patient contact (3.7%), and 588 referrals placed on hold at the patient’s request (3.7%). The referral coordination team covered approximately 90% of outpatient operational hours and successfully contacted 12,713 referred patients (80.0%) within nine minutes of referral entry.

Conclusions

Redesigning outpatient visit closure to include structured, EMR-embedded disposition planning substantially improved the documentation, execution, and reliability of inter-clinic outpatient referrals. By converting informal referral decisions into actionable system processes supported by real-time operational follow-up, the intervention strengthened continuity-of-care processes and improved referral closure without disrupting clinic operations. Structured outpatient disposition represents a scalable strategy to reduce care fragmentation and enhance coordination in high-volume outpatient settings.

## Introduction

Problem description

In our hospital, outpatient visit closure lacked a standardized disposition process. At the conclusion of outpatient encounters, clinicians frequently advised patients to return for follow-up or to consult another outpatient speciality; however, these decisions were often communicated verbally and not consistently documented or operationalized in the electronic medical record (EMR).

As a result, referrals between outpatient clinics and planned follow-up visits (self-referrals) were largely invisible to the system. Baseline data showed that fewer than two inter-clinic outpatient referrals per week were formally recorded in the EMR, despite high outpatient volumes and frequent cross-speciality clinical handoffs. There was no reliable mechanism to confirm whether referred consultations occurred, whether follow-up visits were scheduled, or whether clinical feedback returned to the initiating clinician.

The lack of a structured outpatient disposition planning process resulted in operational failures that impaired care continuity and system performance. In the absence of coordinated follow-up, patients were frequently lost to follow-up or required to self-manage care transitions. Inter-clinic referrals were inconsistently completed, as they relied on patient action rather than a standardized, system-led workflow. This lack of structure also contributed to operational inefficiencies, including underutilized clinic capacity and preventable leakage to external providers. Moreover, the organization was unable to systematically monitor or improve referral performance because referral activity lacked visibility, hindering both oversight and accountability.

Overall, these gaps reflected a process failure at the point of outpatient disposition, where visit closure functioned as an administrative endpoint rather than a clinical and operational handoff. Addressing this failure required redesigning how outpatient encounters are concluded, ensuring that referral and follow-up decisions are explicitly captured, actionable, and supported by the organization.

Available knowledge

Multiple studies have shown that structured referral systems and improved handoff communication enhance continuity of care and improve clinical outcomes [1]. Electronic referral platforms embedded in the EHR (e-referrals) improve the completeness of referral information, appropriateness of referrals, and pre-visit communication between referring clinicians and specialists [2]. In San Francisco’s safety-net system, the implementation of an electronic specialty referral system led to more complete referral information, improved triage, and significantly shorter wait times for routine speciality appointments compared to paper-based referrals [2].

Standardized referral templates also increase the proportion of referrals containing key clinical data and reduce inappropriate or low-value referrals. A national survey in the Veterans Health Administration found that use of such templates was associated with better completeness and clarity of referrals as perceived by specialists [3]. Guidelines for health IT-enabled referral processes recommend standardized order sets and structured formats to minimize variation and ensure key information is captured [4].

Continuity of care itself is strongly associated with better clinical outcomes. Systematic reviews consistently show that higher continuity in outpatient care is linked to fewer avoidable hospitalizations for ambulatory care-sensitive conditions [5]. Among patients with chronic diseases such as diabetes and hypertension, higher continuity is associated with improved delivery of recommended care processes and lower rates of complications, service use, and mortality [6]. While effects on intermediate risk-factor control are more mixed, continuity has been shown to improve long-term outcomes and patient safety [6]. Additionally, greater continuity with a usual doctor, whether a generalist or specialist, is associated with lower all-cause mortality and higher patient satisfaction across health systems [7,8].

This evidence supports the conclusion that closing the referral loop-through electronic platforms, structured templates, and systematic feedback-preserves patients within a continuous care pathway. This in turn contributes to the broader benefits associated with improved continuity, including reduced hospitalizations, lower mortality, and better patient-reported outcomes [1-8].

Rationale

In our hospital, referrals between outpatient clinics were historically informal, poorly documented, and rarely tracked in the EMR. This lack of a structured referral process created gaps in continuity of care and resulted in low visibility of inter-clinic handoffs. We hypothesized that embedding a standardized referral order into the EMR, coupled with operational support, would increase documented referrals and allow the organization to coordinate follow-up more reliably. This hypothesis was grounded in principles from successful interventions in the literature: the use of standardized referral templates [3,4], workflow integration, and feedback mechanisms to sustain clinician engagement [1,2].

Rapid operational follow-up was prioritized due to evidence that delays in scheduling are a known failure point that undermines referral completion and frustrates both patients and providers [9]. Our call centre was integrated into the new workflow to ensure timely outreach to referred patients.

We anticipated that making referrals making visible, standardized, and operationally actionable would convert previously informal actions into measurable system outputs. This would enable real-time monitoring, process accountability, and improved continuity. 

Specific aims

The objective of this quality improvement initiative was to improve the reliability, visibility, and execution of outpatient disposition and referral processes, with a focus on system performance rather than direct clinical outcomes. This quality improvement project aims to establish a standardised electronic referral process for outpatient inter-clinic referrals to improve care coordination and follow-up. Specifically, we intend to increase documented inter-clinic referrals from a baseline of fewer than two per week to at least 500 per week within 16 weeks, and thereafter sustain an average of at least 400 per week for three consecutive months. In tandem, we aim to ensure prompt patient follow-up by contacting over 80% of referred patients within 10 minutes of referral placement. To support continuous improvement and oversight, the project will also implement a real-time dashboard to monitor referral volumes, completion rates, and responsiveness.

## Materials and methods

Context and problem alignment

This quality improvement project was conducted at a large tertiary academic hospital with multiple outpatient speciality clinics. The institution operated a mature electronic medical record (EMR) system; however, prior to this intervention, the end-of-visit disposition for OPD patients was not standardised. Specifically, referrals from one outpatient clinic to another and follow-up (self-referral) decisions were poorly defined, inconsistently documented, and largely invisible at the system level.

While inpatient-to-clinic and emergency-to-clinic referrals were governed by established workflows, OPD-to-OPD referrals and follow-up dispositions were unmanaged. Clinicians frequently advised patients verbally to book with another clinic or come back for follow-up, without a structured mechanism to ensure execution, tracking, or closed-loop communication. As a result, only a negligible number of OPD-to-OPD referrals were formally recorded in the EMR, representing a significant gap in continuity of care, patient safety, and operational reliability.

Hospital leadership identified this gap as a system-level failure occurring at the point of outpatient department (OPD) disposition, with far-reaching implications including fragmented care, missed follow-up opportunities, delayed diagnoses, and patient leakage. In response, a multidisciplinary improvement team, comprising outpatient physicians, administrative leaders, call centre staff, and IT specialists, was convened to redesign the OPD disposition planning process. Particular emphasis was placed on two critical disposition pathways: referrals to colleagues, representing inter-physician referrals, and follow-up appointments within the same service, denoting self-referrals.

As this work constituted a hospital-wide quality improvement initiative, formal research ethics approval was not required; however, the project was reviewed and authorised through the hospital’s quality governance committee.

Intervention, disposition-based referral and follow-up redesign

In May 2025, we implemented a multi-component intervention centred on standardising OPD disposition planning, with electronic referral functionality serving as the enabling mechanism.

Standardised OPD Disposition and Referral Policy

A new policy mandated that all OPD referrals to other clinics and planned follow-up visits must be explicitly documented as part of the OPD disposition. Informal verbal referrals or undocumented follow-up advice were discouraged. Leadership reinforced this policy through departmental meetings and official communications, emphasising disposition clarity as a patient safety and continuity-of-care requirement.

EMR-Embedded Disposition-Based Referral Order

A dedicated electronic medical record (EMR) order, titled “Outpatient Referral to Another Clinic,” was developed and seamlessly embedded within the OPD workflow. This order mandated clinicians to specify key referral details, including the target clinic, the clinical reason for the referral, and the assigned priority level, thereby standardising the referral process and enhancing clarity, accountability, and coordination across services.

This structured order operationalised the “referral to colleagues” disposition state, ensuring standardisation, completeness, and traceability. Entry of the order automatically triggered downstream activation.

Workflow Integration and Call Centre Activation

To achieve closed-loop execution, the referral order generated an automated task for the central call centre. Call centre staff contacted patients promptly, typically within minutes, to schedule the referred appointment or follow-up visit. During the initial rollout phase, call centre staffing was temporarily increased to accommodate higher activation volumes.

Staff Training and Clinical Engagement

All outpatient clinicians and relevant administrative staff received focused training on the OPD disposition framework, including the appropriate use of referral and follow-up orders, as well as the delivery of consistent patient messaging-for example, informing patients that “our team will contact you shortly to arrange your appointment.” To further support adoption and ensure early workflow challenges were addressed, referral champions were designated within each department to reinforce compliance and provide frontline guidance during implementation.

Monitoring, Feedback, and Iterative Refinement

A real-time referral dashboard was developed to monitor utilisation by clinic and physician. Weekly summary reports were shared with department heads, highlighting referral volumes, adoption patterns, and unresolved referrals. This feedback loop supported accountability and rapid troubleshooting. Minor refinements to the EMR form and call centre scripts were made during the first month, while the core disposition-based process remained unchanged. The intervention was implemented hospital-wide simultaneously to avoid parallel workflows and confusion.

Study of the intervention

We employed a pre-post interrupted time series design to evaluate the impact of the intervention. Baseline data were collected for 12 weeks prior to implementation, during which OPD referrals occurred on an ad hoc basis. Post-intervention performance was tracked weekly for 35 weeks.

Statistical process control (SPC) methods were employed to evaluate changes over time, with weekly referral counts plotted using Individuals (I) control charts accompanied by moving ranges. This approach enabled the detection of genuine process shifts and facilitated the assessment of sustainability. The analysis was structured into three distinct phases. Phase 1 encompassed the baseline period (weeks 1 to 12), Phase 2 captured the transition and adoption period (weeks 13 to 19), and Phase 3 represented the sustained implementation of the new process (weeks 20 to 34).

Phase 3 ended at week 34 to allow sufficient post-intervention stability prior to analysis. Control limits were recalculated in Phase 3 following demonstration of special-cause variation. Statistical process control (SPC) was selected over simple pre-post comparisons to better reflect real-world QI dynamics and sustained change. Qualitative feedback from clinicians and call centre staff was reviewed regularly to contextualise quantitative findings and guide iterative improvements.

Measures

The primary outcome of the initiative was the weekly number of OPD-to-OPD referrals documented in the electronic medical record (EMR), serving as a direct measure of uptake for the disposition-based referral process. Secondary outcomes included the timeliness of referral activation, defined as the interval between referral order entry and the first outbound patient contact, and referral outcomes, which were categorised as same-day access, scheduled appointment, patient cancellation, or incomplete. Clinician adoption rate was also assessed, measured by the proportion of outpatient clinicians who utilised the referral order at least once during the study period. Additionally, process quality was evaluated through periodic audits of referral completeness, focusing on the inclusion of referral reasons and supporting clinical information. All data were extracted from the EMR and call centre systems and analysed using Microsoft Excel with quality improvement statistical process control (SPC) macros.

Analysis

Descriptive statistics were used for secondary outcomes. No hypothesis testing was performed, as this was a full-population QI intervention. Effectiveness was interpreted based on temporal association, SPC signals, and sustained process stability, consistent with best practices in quality improvement evaluation.

## Results

Referral volume increase

**Figure 1:**
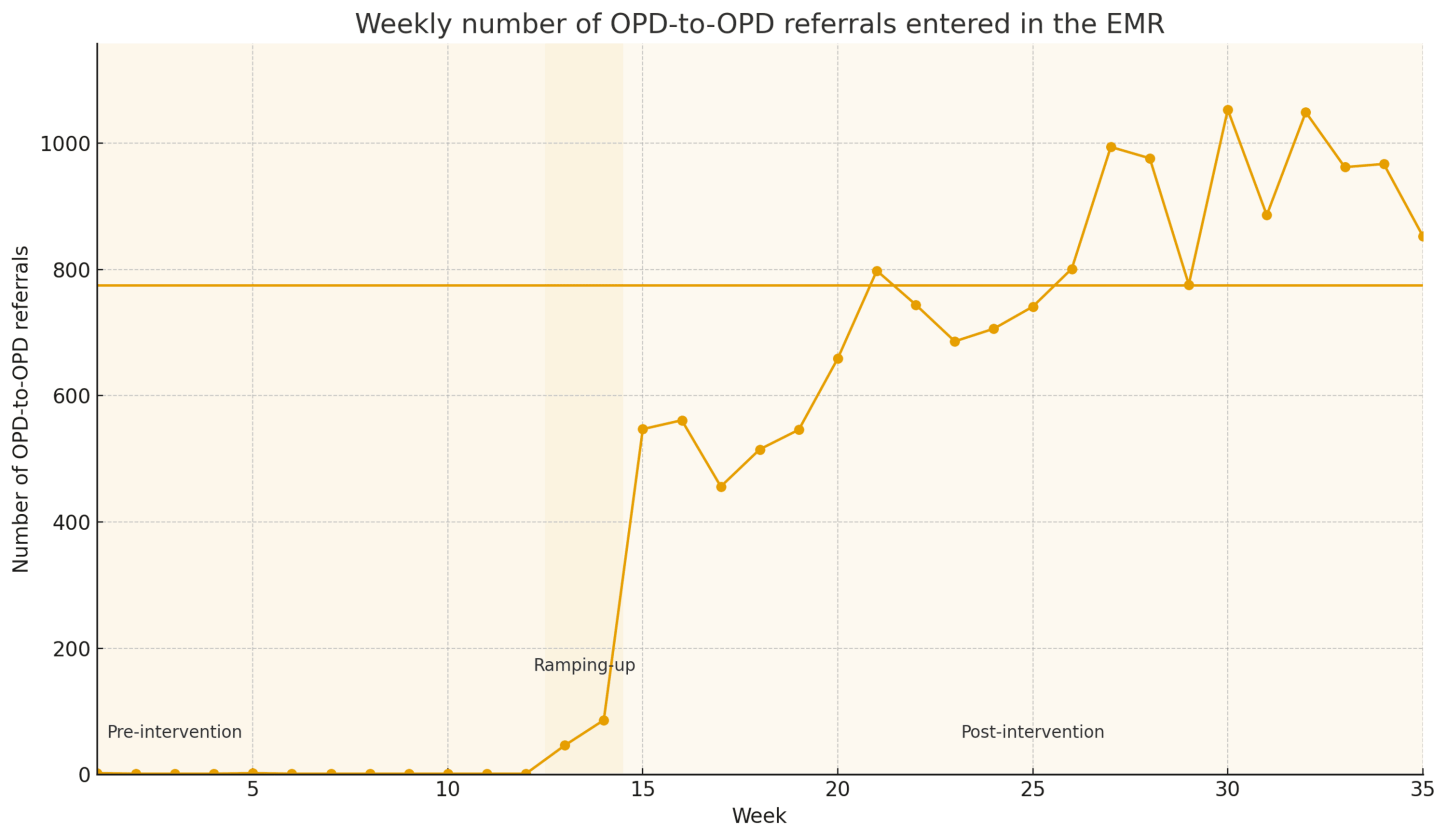
Weekly OPD-to-OPD Referral Volume Before and After Implementation of the Electronic Closed-Loop Referral Pathway

The intervention resulted in an immediate and sustained increase in documented inter-clinic outpatient referrals. During the 12-week baseline period, the average number of documented referrals was 1.17 per week, reflecting minimal use of the previous referral workflow. In the first week after implementation, referral volume rose sharply to 342. By week five, referrals approached 800 per week. A stable plateau was reached by the second month. During Phase 3 (weeks 20-34), the mean number of referrals was 853.2 per week, with a weekly range between approximately 750 and 950. This sustained performance met both Aim #2 (≥500 referrals/week by 16 weeks) and Aim #3 (sustain ≥400/week for ≥3 months). Figure 1* *displays the weekly referral counts across the baseline and intervention periods.

**Figure 2:**
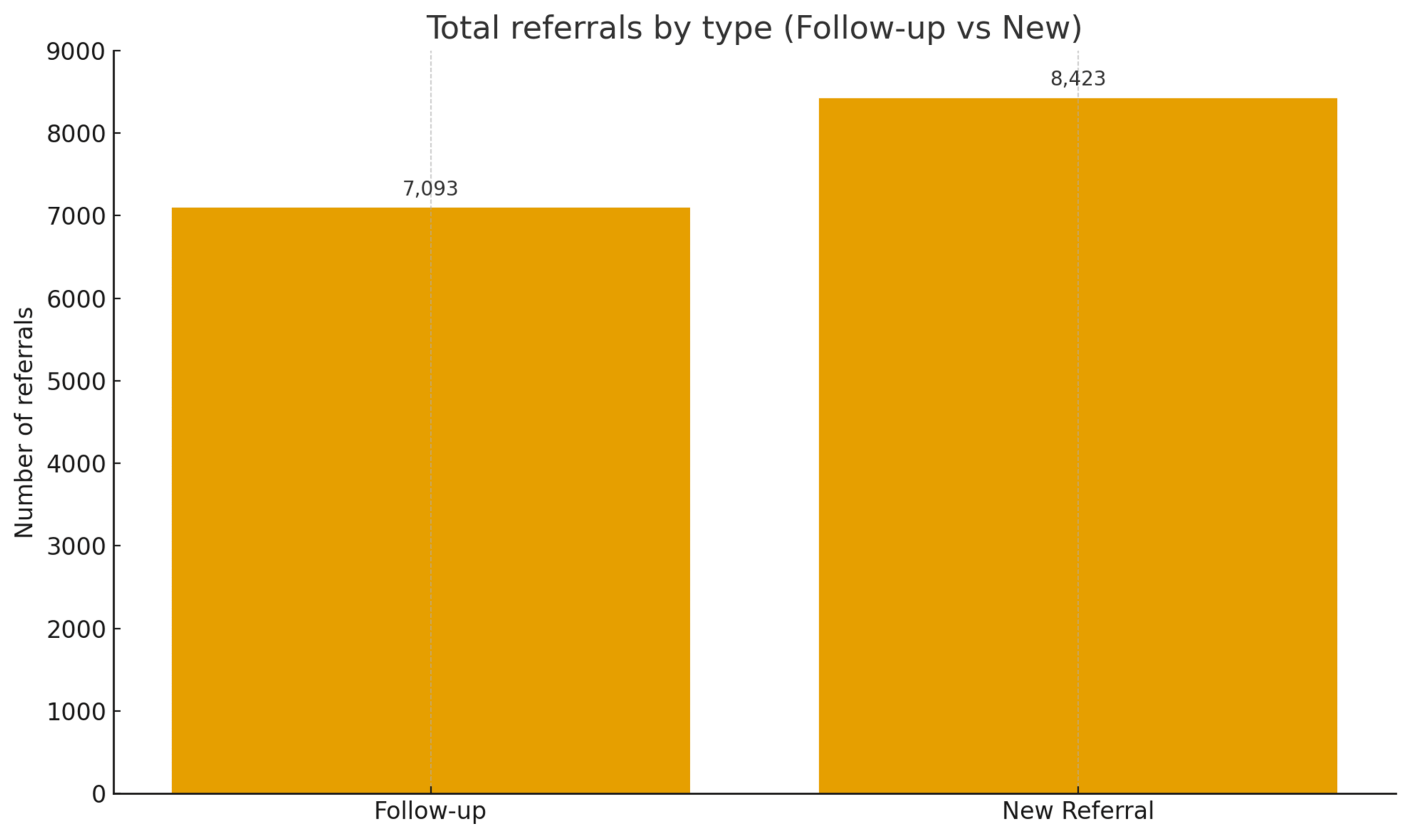
Distribution of OPD-to-OPD Referral Types Over the 35-Week Post-Implementation Period

Over 35 weeks following implementation, a total of 15,891 inter-clinic referrals were logged. Of these, 8,423 (53%) were new consultations, representing patients being referred to a speciality clinic for the first time. The remaining 7,093 (47%) were follow-up referrals, typically for structured transitions of care or continued outpatient management across departments. Figure 2 shows the distribution between new and follow-up referral types.

Referral Outcomes (Appointment Status)

**Figure 3:**
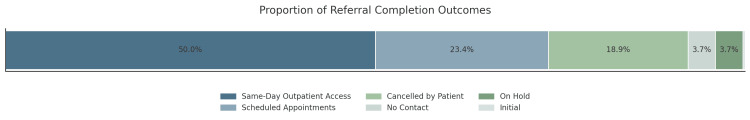
Referral Completion Outcome

The outcomes of those 15,891 referrals are broken down in Figure 3 and summarised here.

Scheduled Appointments

3,726 referrals (23.4%) led to an outpatient clinic appointment being booked for the patient.

*Direct Admissions* 

Same-day outpatient access occurred in 7,948 referrals (50.0%), indicating that half of all referrals were completed with same-day specialist intake.

*Cancelled by Patien*t

3,006 referrals (18.9%) were scheduled, but then the patient cancelled the appointment (or declined the referral).

No Contact/On Hold

1,169 referrals (7.4%) did not result in immediate scheduling. Specifically, 581 patients (3.7%) did not answer or respond to repeated call attempts (not responding), and 588 (3.7%) were put on hold (e.g., patient asked to postpone scheduling, or needed to decide). These remained open in the system for follow-up.

Initial State

Forty-two referrals (0.3%) were in an initial state at analysis time (just placed, awaiting processing).

Combining the first two outcome categories, 73.5% of all referrals achieved a next step in care. This indicates a strong closure of the loop. The roughly one-quarter of referrals that did not complete were largely patient-driven cancellations or inability to reach patients. This is an area identified for further improvement (e.g., enhanced patient education around the importance of the referral, or updated contact information processes).

Time to First Patient Contact (Secondary Outcome)

The referral coordination team operated during 90% of outpatient clinic hours. Within this coverage window, the team successfully contacted 80% of referred patients within nine minutes of referral entry. This immediate engagement likely contributed to the high rate of successful scheduling, and qualitative feedback indicated that patients perceived this responsiveness positively, often noting the unexpected speed with which the hospital reached out.

Provider Adoption and Referral Patterns (Secondary Outcome)

**Figure 4:**
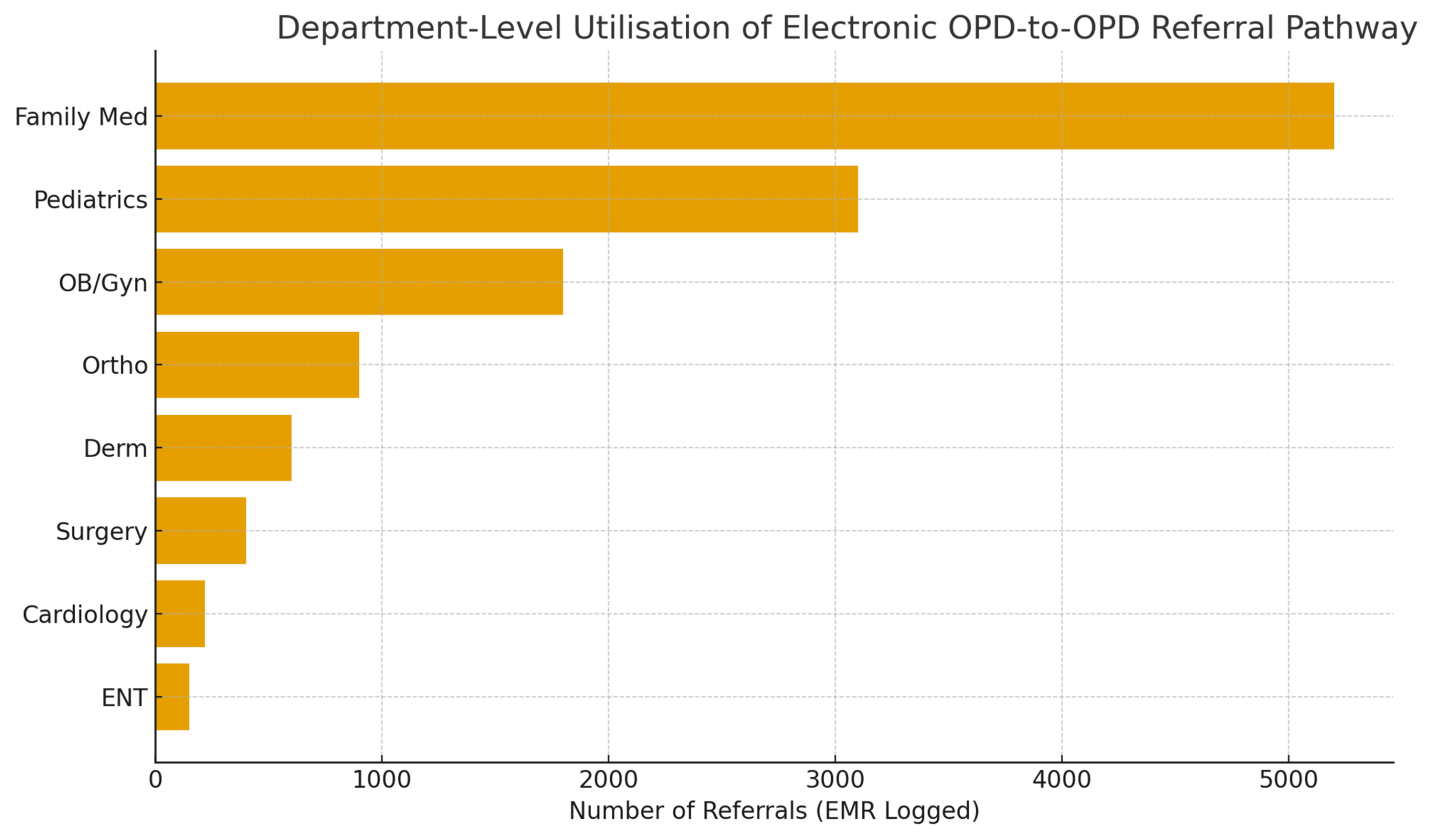
Analysis of Provider-Level Usage Revealed Substantial Heterogeneity in Adoption of the Electronic Referral Pathway.

Referral activity was concentrated among a small proportion of providers. Analysis showed that roughly 10% of outpatient clinicians used the referral order at least once during the 35 weeks. Among these, the top ten referrers accounted for a large share of total volume. High adoption rates were observed in family medicine, pediatrics, obstetrics, and selected surgical specialities. Figure 4 shows the distribution of referral volume by individual provider.

**Figure 5:**
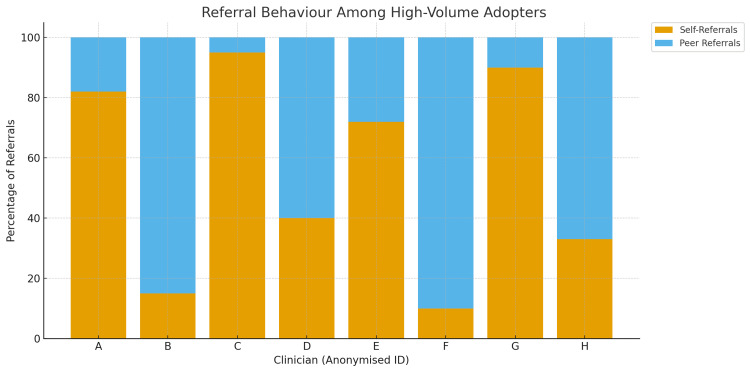
Referral Behavior Among High-Volume Adopters

A clearer pattern emerges in* *Figure 5, which classifies the referral behavior of high-volume adopters into two dominant patterns. The continuity-focused pattern is characterized by very high proportions of self-referrals-often exceeding 80-100%-reflecting structured follow-up planning for chronic disease management, antenatal care, and pediatrics. In contrast, clinicians with a consultation-focused profile displayed high peer-referral ratios, formalizing inter-speciality transitions but risking a shift of continuity away from primary care if not coupled with planned re-engagement.

The two visualisations highlight both the strengths and gaps of the implementation. Among adopters, the system substantially improved continuity and closed-loop coordination; however, the sharp drop-off beyond the top decile of users demonstrates that a large proportion of outpatient clinicians continued relying on parallel, informal pathways. This uneven adoption remains a key target for subsequent improvement cycles and institutional policy alignment.

In summary, the intervention produced a marked improvement in the outpatient referral pathway. Table 1 summarises the key pre and post-intervention metrics. Although overall physician adoption of the electronic referral system remained limited at approximately 10%, the referrals submitted through the platform were handled efficiently and yielded clear downstream benefits. These included a substantial rise in completed referral loops, rapid patient contact, and timely scheduling without evidence of unintended operational burden. No clinics reported being overwhelmed by referral-related demand; most noted that the referred patients were those who required evaluation regardless of the new process. A small number of services adjusted their scheduling templates to create dedicated referral capacity, an operational refinement that is being managed administratively.

**Table 1:** Comparison of Key Referral Process Outcomes Before and After Implementation.

Outcome Measures	Baseline (Before)	After Intervention
Documented OPD-to-OPD referrals	~1 per week (ad hoc)	~853 per week (mean)
Total referrals in 35 weeks	~40 (estimated)	15,891
% of referrals completed (appointment or admission)	N/A (process not tracked)	73.50%
Time to contact patient	N/A (no process)	80% within 9 minutes

## Discussion

This quality improvement initiative addressed a critical operational and clinical gap in outpatient care delivery: the absence of a standardized, system-supported disposition process at the point of outpatient visit closure. Prior to the intervention, referral and follow-up decisions were frequently communicated verbally and were not consistently captured or operationalized in the electronic medical record (EMR). This resulted in poor visibility of inter-clinic referrals, unreliable execution, fragmented continuity of care, and limited operational oversight. By embedding a structured inter-clinic outpatient referral pathway into the EMR and linking it to real-time operational follow-up, the initiative transformed outpatient disposition from an administrative endpoint into an actionable clinical and operational handoff.

Principal findings

The intervention resulted in a rapid and sustained increase in documented inter-clinic outpatient referrals, rising from fewer than two referrals per week at baseline to more than 800 referrals per week within four months of implementation. This level of performance was sustained for over nine months, indicating durable process change rather than a transient improvement. More than 70% of referrals resulted in a completed next step, either a scheduled outpatient appointment or same-day access, demonstrating effective closure of the referral loop.

Operational responsiveness was a key contributor to success. The referral coordination team covered approximately 90% of outpatient operational hours and successfully contacted 80% of referred patients within nine minutes of referral entry, meeting the predefined performance target. Delays in referral scheduling are a known cause of referral failure and patient dissatisfaction, and rapid outreach has been shown to improve referral completion and patient trust in the system [10,11]. The high contact rate observed in this initiative likely contributed to the relatively low proportion of unresolved referrals.

Although adoption was limited to a subset of clinicians (approximately 10%), those who adopted the referral tool used it consistently and at high volume. Referral behavior clustered into two dominant patterns: continuity-focused use (planned follow-up or self-referral within the same service) and consultation-focused use (referral to another outpatient speciality). This finding suggests that the referral pathway was flexible enough to support different clinical intents while maintaining system visibility and accountability.

Comparison with prior work

These findings are consistent with prior literature demonstrating that electronic referral systems embedded within the EMR improve referral documentation, appropriateness, and completion. The San Francisco safety-net eReferral program demonstrated significant improvements in referral quality, triage efficiency, and wait times following implementation of an electronic speciality referral system [12]. Similarly, studies within the Veterans Health Administration and other health systems have shown that standardized referral templates improve the completeness of referral information and reduce inappropriate or low-value referrals [13,14].

Most previously published referral interventions focus on primary care-to-speciality transitions. This initiative extends that work by addressing inter-clinic outpatient referrals across multiple specialities within a single institution and by embedding referral capture directly into outpatient disposition planning. Failures at visit closure, rather than at referral receipt, represent a common but underrecognized source of continuity breakdown, and this intervention directly targeted that failure point.

Continuity of care is strongly associated with improved outcomes, including fewer avoidable hospitalizations, reduced emergency department utilization, and lower mortality [15-17]. Systematic reviews consistently demonstrate that patients who experience higher continuity with outpatient providers have better long-term outcomes, even when improvements in short-term surrogate markers are modest [16]. By ensuring that follow-up and referral decisions were explicitly captured, tracked, and operationalized, this intervention supported continuity both within services and across outpatient specialities.

Strengths and implications

A major strength of this initiative was the alignment of clinical decision-making with operational execution. Referral intent entered by clinicians was immediately translated into system action through call centre activation, reducing reliance on patient initiative and minimizing loss to follow-up. Embedding the referral order within routine workflows minimized additional cognitive or administrative burden. The development of a live referral dashboard enabled real-time monitoring, transparency, and performance oversight-capabilities that were previously absent. This visibility allowed leadership and operational teams to identify trends, assess capacity utilization, and respond proactively to demand. Importantly, no receiving clinics reported operational overload, and some adjusted scheduling templates to better accommodate internal referral demand, suggesting improved alignment between service capacity and clinical need. These findings have broader implications for outpatient care redesign. Improving continuity and referral reliability does not necessarily require new infrastructure but rather intentional redesign of decision points, particularly visit disposition, and alignment between EMR workflows and operational support.

Limitations

This project has several limitations. Adoption was concentrated among a subset of clinicians, which may limit generalizability and indicate the need for further engagement strategies. Patient-reported outcomes and satisfaction were not formally measured. Downstream clinical outcomes and cost impact were also not evaluated. Approximately one-quarter of referrals did not result in scheduled visits, largely due to patient cancellations or inability to establish contact, representing opportunities for further refinement. Finally, this was a single-centre initiative conducted in a tertiary hospital with centralized scheduling infrastructure, which may limit transferability to other settings.

Future directions

Future improvement cycles will focus on expanding clinician adoption, standardizing referral criteria, and integrating patient preference capture at the time of referral. Planned evaluations include referral completion timelines, downstream clinical outcomes, patient experience measures, and service utilization impact. The model may also be extended to include external referrals and digital patient self-scheduling to further enhance continuity of care.

## Conclusions

This quality improvement initiative demonstrated that redesigning outpatient visit closure to include structured, EMR-embedded disposition planning can substantially improve the visibility, execution, and reliability of inter-clinic outpatient referrals. By converting previously informal referral and follow-up decisions into standardized, actionable system processes supported by real-time operational follow-up, our institution achieved a rapid and sustained increase in documented referrals, timely patient engagement, and high rates of referral closure.

The findings highlight the importance of addressing system failures at the point of outpatient disposition, where clinical intent is often lost without structured capture and operational support. Embedding referral pathways into routine workflows, coupled with responsive coordination mechanisms, enabled improved continuity of care processes without adding significant burden to clinicians or disrupting receiving services. While adoption was initially limited to a subset of providers, high-impact use among adopters and sustained performance over time suggest that structured referral systems can meaningfully strengthen outpatient care coordination. This model offers a scalable approach for healthcare organizations seeking to reduce fragmentation, improve continuity, and enhance operational oversight of outpatient referrals through targeted workflow redesign rather than new infrastructure.

Further work is needed to expand adoption, assess patient-reported outcomes, and evaluate downstream clinical and utilization impacts. Nonetheless, this initiative demonstrates that deliberate redesign of outpatient disposition processes represents a practical and effective strategy for closing the referral loop and supporting continuity of care in complex outpatient environments.
